# Supplementary material for: HIVprotI: an integrated web based platform for prediction and design of HIV proteins inhibitors
Source: J Cheminform. 2018 Mar 9;10:12. doi: 10.1186/s13321-018-0266-y (PMC5845081; doi:10.1186/s13321-018-0266-y)
Supplement: Supplementary file 2 — Additional file 2. Source code of HIVProtI web server. [file 13321_2018_266_MOESM2_ESM.zip › HIVprotI_Source-code/hivprotI/weka-3-6-6/doc/weka/gui/AttributeVisualizationPanel.html]

AttributeVisualizationPanel


---


|  |  |  |
| --- | --- | --- |
| **Overview** \| **Package** \| **Class** \| **Tree** \| **Deprecated** \| **Index** \| **Help** \| **Weka's home** | |  |
| **PREV CLASS**   **NEXT CLASS** | **FRAMES**    **NO FRAMES**     **All Classes** |  |
| SUMMARY: NESTED \| FIELD \| CONSTR \| METHOD | DETAIL: FIELD \| CONSTR \| METHOD |  |


---


## weka.gui Class AttributeVisualizationPanel

```
java.lang.Object
  java.awt.Component
      java.awt.Container
          javax.swing.JComponent
              javax.swing.JPanel
                  weka.gui.visualize.PrintablePanel
                      weka.gui.AttributeVisualizationPanel
```

**All Implemented Interfaces:**: java.awt.image.ImageObserver, java.awt.MenuContainer, java.io.Serializable, javax.accessibility.Accessible, PrintableHandler

---

``` public class AttributeVisualizationPanel extends PrintablePanel ```

Creates a panel that shows a visualization of an
attribute in a dataset. For nominal attribute it
shows a bar plot, with each bar corresponding to
each nominal value of the attribute with its height
equal to the frequecy that value appears in the
dataset. For numeric attributes, it displays a
histogram. The width of an interval in the
histogram is calculated using Scott's(1979)
method:   
intervalWidth = Max(1, 3.49\*Std.Dev\*numInstances^(1/3))
Then the number of intervals is calculated by:   
intervals = max(1, Math.round(Range/intervalWidth);

**Version:**
:   $Revision: 7589 $

**Author:**
:   Ashraf M. Kibriya (amk14@cs.waikato.ac.nz)

**See Also:**: Serialized Form

---

**Nested Class Summary**

**Nested classes/interfaces inherited from class javax.swing.JComponent**

`javax.swing.JComponent.AccessibleJComponent`

**Nested classes/interfaces inherited from class java.awt.Component**

`java.awt.Component.BaselineResizeBehavior`


**Field Summary**

**Fields inherited from class javax.swing.JComponent**

`TOOL_TIP_TEXT_KEY, UNDEFINED_CONDITION, WHEN_ANCESTOR_OF_FOCUSED_COMPONENT, WHEN_FOCUSED, WHEN_IN_FOCUSED_WINDOW`

**Fields inherited from class java.awt.Component**

`BOTTOM_ALIGNMENT, CENTER_ALIGNMENT, LEFT_ALIGNMENT, RIGHT_ALIGNMENT, TOP_ALIGNMENT`

**Fields inherited from interface java.awt.image.ImageObserver**

`ABORT, ALLBITS, ERROR, FRAMEBITS, HEIGHT, PROPERTIES, SOMEBITS, WIDTH`


**Constructor Summary**

`AttributeVisualizationPanel()`
  
          Constructor - If used then the class will not show the class selection
combo box.

`AttributeVisualizationPanel(boolean showColouringOption)`
  
          Constructor.


| **Method Summary** | |
| --- | --- |
| `void` | `calcGraph(int panelWidth, int panelHeight)`             Recalculates the barplot or histogram to display, required usually when the attribute is changed or the component is resized. |
| `javax.swing.JComboBox` | `getColorBox()`             Returns the class selection combo box if the parent component wants to place it in itself or in some component other than this component. |
| `int` | `getColoringIndex()`             Get the coloring (class) index for the plot |
| `java.lang.String` | `getToolTipText(java.awt.event.MouseEvent ev)`             Returns "<nominal value> [<nominal value count>]" if displaying a bar plot and mouse is on some bar. |
| `static void` | `main(java.lang.String[] args)`             Main method to test this class from command line |
| `void` | `paintComponent(java.awt.Graphics g)`             Paints this component |
| `void` | `setAttribute(int index)`             Tells the panel which attribute to visualize. |
| `void` | `setColoringIndex(int ci)`             Set the coloring (class) index for the plot |
| `void` | `setInstances(Instances newins)`             Sets the instances for use |

**Methods inherited from class weka.gui.visualize.PrintablePanel**

`getSaveDialogTitle, getWriter, getWriters, getXScale, getYScale, saveComponent, setSaveDialogTitle, setScale`

**Methods inherited from class javax.swing.JPanel**

`getAccessibleContext, getUI, getUIClassID, setUI, updateUI`

**Methods inherited from class javax.swing.JComponent**

`addAncestorListener, addNotify, addVetoableChangeListener, computeVisibleRect, contains, createToolTip, disable, enable, firePropertyChange, firePropertyChange, firePropertyChange, getActionForKeyStroke, getActionMap, getAlignmentX, getAlignmentY, getAncestorListeners, getAutoscrolls, getBaseline, getBaselineResizeBehavior, getBorder, getBounds, getClientProperty, getComponentPopupMenu, getConditionForKeyStroke, getDebugGraphicsOptions, getDefaultLocale, getFontMetrics, getGraphics, getHeight, getInheritsPopupMenu, getInputMap, getInputMap, getInputVerifier, getInsets, getInsets, getListeners, getLocation, getMaximumSize, getMinimumSize, getNextFocusableComponent, getPopupLocation, getPreferredSize, getRegisteredKeyStrokes, getRootPane, getSize, getToolTipLocation, getToolTipText, getTopLevelAncestor, getTransferHandler, getVerifyInputWhenFocusTarget, getVetoableChangeListeners, getVisibleRect, getWidth, getX, getY, grabFocus, isDoubleBuffered, isLightweightComponent, isManagingFocus, isOpaque, isOptimizedDrawingEnabled, isPaintingForPrint, isPaintingTile, isRequestFocusEnabled, isValidateRoot, paint, paintImmediately, paintImmediately, print, printAll, putClientProperty, registerKeyboardAction, registerKeyboardAction, removeAncestorListener, removeNotify, removeVetoableChangeListener, repaint, repaint, requestDefaultFocus, requestFocus, requestFocus, requestFocusInWindow, resetKeyboardActions, reshape, revalidate, scrollRectToVisible, setActionMap, setAlignmentX, setAlignmentY, setAutoscrolls, setBackground, setBorder, setComponentPopupMenu, setDebugGraphicsOptions, setDefaultLocale, setDoubleBuffered, setEnabled, setFocusTraversalKeys, setFont, setForeground, setInheritsPopupMenu, setInputMap, setInputVerifier, setMaximumSize, setMinimumSize, setNextFocusableComponent, setOpaque, setPreferredSize, setRequestFocusEnabled, setToolTipText, setTransferHandler, setVerifyInputWhenFocusTarget, setVisible, unregisterKeyboardAction, update`

**Methods inherited from class java.awt.Container**

`add, add, add, add, add, addContainerListener, addPropertyChangeListener, addPropertyChangeListener, applyComponentOrientation, areFocusTraversalKeysSet, countComponents, deliverEvent, doLayout, findComponentAt, findComponentAt, getComponent, getComponentAt, getComponentAt, getComponentCount, getComponents, getComponentZOrder, getContainerListeners, getFocusTraversalKeys, getFocusTraversalPolicy, getLayout, getMousePosition, insets, invalidate, isAncestorOf, isFocusCycleRoot, isFocusCycleRoot, isFocusTraversalPolicyProvider, isFocusTraversalPolicySet, layout, list, list, locate, minimumSize, paintComponents, preferredSize, printComponents, remove, remove, removeAll, removeContainerListener, setComponentZOrder, setFocusCycleRoot, setFocusTraversalPolicy, setFocusTraversalPolicyProvider, setLayout, transferFocusBackward, transferFocusDownCycle, validate`

**Methods inherited from class java.awt.Component**

`action, add, addComponentListener, addFocusListener, addHierarchyBoundsListener, addHierarchyListener, addInputMethodListener, addKeyListener, addMouseListener, addMouseMotionListener, addMouseWheelListener, bounds, checkImage, checkImage, contains, createImage, createImage, createVolatileImage, createVolatileImage, dispatchEvent, enable, enableInputMethods, firePropertyChange, firePropertyChange, firePropertyChange, firePropertyChange, firePropertyChange, getBackground, getBounds, getColorModel, getComponentListeners, getComponentOrientation, getCursor, getDropTarget, getFocusCycleRootAncestor, getFocusListeners, getFocusTraversalKeysEnabled, getFont, getForeground, getGraphicsConfiguration, getHierarchyBoundsListeners, getHierarchyListeners, getIgnoreRepaint, getInputContext, getInputMethodListeners, getInputMethodRequests, getKeyListeners, getLocale, getLocation, getLocationOnScreen, getMouseListeners, getMouseMotionListeners, getMousePosition, getMouseWheelListeners, getName, getParent, getPeer, getPropertyChangeListeners, getPropertyChangeListeners, getSize, getToolkit, getTreeLock, gotFocus, handleEvent, hasFocus, hide, imageUpdate, inside, isBackgroundSet, isCursorSet, isDisplayable, isEnabled, isFocusable, isFocusOwner, isFocusTraversable, isFontSet, isForegroundSet, isLightweight, isMaximumSizeSet, isMinimumSizeSet, isPreferredSizeSet, isShowing, isValid, isVisible, keyDown, keyUp, list, list, list, location, lostFocus, mouseDown, mouseDrag, mouseEnter, mouseExit, mouseMove, mouseUp, move, nextFocus, paintAll, postEvent, prepareImage, prepareImage, remove, removeComponentListener, removeFocusListener, removeHierarchyBoundsListener, removeHierarchyListener, removeInputMethodListener, removeKeyListener, removeMouseListener, removeMouseMotionListener, removeMouseWheelListener, removePropertyChangeListener, removePropertyChangeListener, repaint, repaint, repaint, resize, resize, setBounds, setBounds, setComponentOrientation, setCursor, setDropTarget, setFocusable, setFocusTraversalKeysEnabled, setIgnoreRepaint, setLocale, setLocation, setLocation, setName, setSize, setSize, show, show, size, toString, transferFocus, transferFocusUpCycle`

**Methods inherited from class java.lang.Object**

`equals, getClass, hashCode, notify, notifyAll, wait, wait, wait`

**Constructor Detail**

### AttributeVisualizationPanel

```
public AttributeVisualizationPanel()
```

:   Constructor - If used then the class will not show the class selection
    combo box.

---


### AttributeVisualizationPanel

```
public AttributeVisualizationPanel(boolean showColouringOption)
```

:   Constructor.

    **Parameters:**: `showColouringOption` - - should be true if the class selection combo box is to be displayed with the histogram/barplot, or false otherwise. P.S: the combo box is always created it just won't be shown if showColouringOption is false.


**Method Detail**

### setInstances

```
public void setInstances(Instances newins)
```

:   Sets the instances for use

    :   **Parameters:**: `newins` - a set of Instances

---


### getColorBox

```
public javax.swing.JComboBox getColorBox()
```

:   Returns the class selection combo box if the parent component wants to
    place it in itself or in some component other than this component.

---


### getColoringIndex

```
public int getColoringIndex()
```

:   Get the coloring (class) index for the plot

    :   **Returns:**: an `int` value

---


### setColoringIndex

```
public void setColoringIndex(int ci)
```

:   Set the coloring (class) index for the plot

    :   **Parameters:**: `ci` - an `int` value

---


### setAttribute

```
public void setAttribute(int index)
```

:   Tells the panel which attribute to visualize.

    :   **Parameters:**: `index` - The index of the attribute

---


### calcGraph

```
public void calcGraph(int panelWidth,
                      int panelHeight)
```

:   Recalculates the barplot or histogram to display, required usually when the
    attribute is changed or the component is resized.

---


### getToolTipText

```
public java.lang.String getToolTipText(java.awt.event.MouseEvent ev)
```

:   Returns "<nominal value> [<nominal value count>]"
    if displaying a bar plot and mouse is on some bar.
    If displaying histogram then it- returns "count <br> [<bars Range>]" if mouse is
      on the first bar.
    - returns "count <br> (<bar's Range>]" if mouse is
      on some bar other than the first one.
    Otherwise it returns ""

    :   **Overrides:**: `getToolTipText` in class `javax.swing.JComponent`
    :   **Parameters:**: `ev` - The mouse event

---


### paintComponent

```
public void paintComponent(java.awt.Graphics g)
```

:   Paints this component

    :   **Overrides:**: `paintComponent` in class `javax.swing.JComponent`
    :   **Parameters:**: `g` - The graphics object for this component

---


### main

```
public static void main(java.lang.String[] args)
```

:   Main method to test this class from command line

    :   **Parameters:**: `args` - The arff file and the index of the attribute to use


---


|  |  |  |
| --- | --- | --- |
| **Overview** \| **Package** \| **Class** \| **Tree** \| **Deprecated** \| **Index** \| **Help** \| **Weka's home** | |  |
| **PREV CLASS**   **NEXT CLASS** | **FRAMES**    **NO FRAMES**     **All Classes** |  |
| SUMMARY: NESTED \| FIELD \| CONSTR \| METHOD | DETAIL: FIELD \| CONSTR \| METHOD |  |


---
